# Morphological, Chemical Surface, and Diffusive Transport Characterizations of a Nanoporous Alumina Membrane

**DOI:** 10.3390/nano5042192

**Published:** 2015-12-05

**Authors:** María I. Vázquez, Virgina Romero, Victor Vega, Javier García, Victor M. Prida, Blanca Hernando, Juana Benavente

**Affiliations:** 1 Department of Applied Physics I, Faculty of Sciences, University of Málaga, E-29071 Málaga, Spain; E-Mails: mvazquez@uma.es (M.I.V.); virgirom@uma.es (V.R.); 2 Department of Physics, Faculty of Sciences, University of Oviedo, E-33007 Oviedo, Spain; E-Mails: vegavictor@uniovi.es (V.V.); jgarcia@physnet.uni-hamburg.de (J.G.); vmpp@uniovi.es (V.M.P.); grande@uniovi.es (B.H.); 3 Institute of Applied Physics, University of Hamburg, 20355-Hamburg, Germany

**Keywords:** nanoporous alumina membrane, SEM analysis, diffusion coefficients, ion transport numbers

## Abstract

Synthesis of a nanoporous alumina membrane (NPAM) by the two-step anodization method and its morphological and chemical surface characterization by analyzing Scanning Electron Microscopy (SEM) micrographs and X-Ray Photoelectron Spectroscopy (XPS) spectra is reported. Influence of electrical and diffusive effects on the NaCl transport across the membrane nanopores is determined from salt diffusion measurements performed with a wide range of NaCl concentrations, which allows the estimation of characteristic electrochemical membrane parameters such as the NaCl diffusion coefficient and the concentration of fixed charges in the membrane, by using an appropriated model and the membrane geometrical parameters (porosity and pore length). These results indicate a reduction of ~70% in the value of the NaCl diffusion coefficient through the membrane pores with respect to solution. The transport number of ions in the membrane pores (Na^+^ and Cl^−^, respectively) were determined from concentration potential measurements, and the effect of concentration-polarization at the membrane surfaces was also considered by comparing concentration potential values obtained with stirred solutions (550 rpm) and without stirring. From both kinds of results, a value higher than 0.05 M NaCl for the feed solution seems to be necessary to neglect the contribution of electrical interactions in the diffusive transport.

## 1. Introduction

Nanoporous ordered structures have gained attention in the last two decades for different applications such as templates for nanoparticles, nanotubes, and nanowires synthesis [1], but also in devices commonly used in nanofluidics, sensors, or drug delivery applications [2,3,4,5]. Silica, alumina, or titania nanotubular structures with regular pore diameters and interpore distances can be used as models systems for fluid transport [6,7,8,9]. Particularly, the analysis of the diffusive transport of molecules or ions through a nanochannel or nanopore associated to a concentration difference of the solutions at both channel ends is basic in practically all these latter applications.

Among the periodic porous nanostructures commonly used in the study of confined diffusive transport, the nanoporous anodic alumina membranes (NPAMs) fabricated via electrochemical anodization of aluminium are of singular interest due to their easy fabrication, high chemical and thermal stability and well-defined geometric structure with accurate nanopore diameters and narrow pore size distribution [10,11,12]. Another point of interest related with the use of NPAMs is the possibility of pore modification by surface coating, which can affect both the size and chemical nature of the pore, this point being of significant importance for their application as biosensors or in biomedical devices due to specific characteristics of the solutions (or solutes) [13]. In fact, the effect on the diffusive transport through nanopores of the electrical membrane-solute (charged molecules or ions) interactions related with the pore surface material as well as its dependence on the pore size and porosity have already been revealed [14]. Moreover, other effects able to affect the diffusive transport of charged molecules and ions such as concentration polarization or the Donnan exclusion effect, this latter in the case of charged solids/electrolyte solutions, should also be considered since they could explain the extremely reduced diffusivity values through nanoporous membranes obtained from indirect measurements (time-resolved fluorescence imaging [4], decoloration by photometric measurements [15], *etc.*). Although these effects have already been demonstrated in previous works by using membrane potentials or radiotracer diffusion measurements [14,16], these experimental techniques are not commonly employed in drug delivery studies, which usually analyze solute flow without paying too much attention to solution stirring or feed solution level and, consequently, to the possible masking effect of concentration polarization or electrical interactions on the obtained results [17,18]. In fact, Bluhm *et al.* [17] does not find practical differences in the diffusion coefficient of different ions through two NPAMs with 20 nm or 200 nm pore radius for 10^−4^ M feed concentration, which was attributed to the electrical double layer (electrical interactions) at the membrane/solution interface. The election of the solution concentration might significantly enlarge the contribution of that effect, taking into account the concentration dependence of the different electrokinetic phenomena [19].

This work describes the synthesis of a NPAM by the two-step anodization method in oxalic acid electrolyte together with its morphological and chemical surface characterizations by SEM and XPS analysis as well as the diffusive transport of salt (NaCl) and ions (Cl^−^ and Na^+^) through the membrane nanopores (pore radii of 23 nm). Salt diffusion measurements were performed with NaCl solutions and a wide range of concentrations (seven values ranging from 0.005 M to 0.4 M), and the analysis of the diffusive permeability *versus* feed concentration curve allows us the estimation of two characteristic electrochemical parameters such as the diffusion coefficient in the nanopores (*D_i_*) and the effective fixed charge in the membrane (*X_ef_*) [20]. This latter parameter also gives valuable information on the minimum solution concentration value necessary to minimize the electrical interaction effect on diffusive transport (C >> *X_ef_*). Moreover, from concentration potentials measurements the ions (Cl^−^ and Na^+^) transport numbers in the alumina membrane (*t*_i_) and their dependence with the average solution concentration were also determined; the low values obtained for the Na^+^ transport number in the alumina membrane confirm its electropositive character, and the effect on *t*_i_ values associated to differences in the solution stirring rate as a result of concentration polarization is also determined. Our results evidence the importance of carefully selecting the experimental conditions, *i.e.*, electrolyte concentration and stirring conditions, for a more adequate characterization of the diffusive ion transport through charged nanoporous membranes.

## 2. Results and Discussion

### 2.1. Morphological and Chemical Surface Characterization

**Figure 1:**
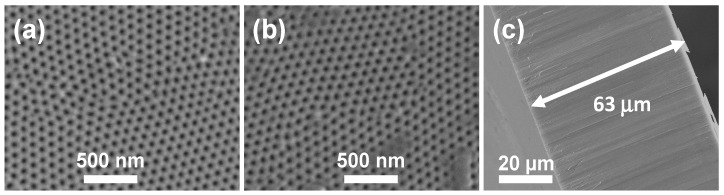
Scanning Electron Microscopy (SEM) micrographs of both membrane surfaces, (**a**) and (**b**), and cross-section (**c**) of the Al-O*x* nanoporous alumina membrane (NPAM).

Morphological characterization of the Al-O*x* membrane was performed by SEM. Figure 1 shows micrographs for the both surfaces of the nanoporous alumina membrane together with its cross-section, where the ordered pore structure and uniform thickness exhibited by the Al-O*x* membrane can be clearly observed. Average pore size, *d*_p_, interpore distance, *D*_int_, and thickness for the Al-O*x* membrane were determined by image analysis of the SEM micrographs and the following average values were obtained: the top surface (a) shows <*r*_p_>_top_ = (23 ± 3) nm, while for the bottom surface (b) <*r*_p_>_bottom_ = (24 ± 3) nm. These values of the pore radii indicate a slight pore widening with respect to the expected value of <*r*_p_> = (17 ± 3) nm, which is due to the chemical etching process in H_3_PO_4_ acidic solution employed to remove the alumina barrier layer at the pore bottom (see Item 3.1). In both cases, the average interpore distance, *D*_int_, takes values of approximately 105 nm, while the total membrane thickness, <∆*x*>, measured at the membrane cross section (c) takes a value of 63 ± 2 μm.

Chemical surface characterization of the Al-O*x* NPAM was carried out by analyzing XPS spectra. Table 1 shows the atomic concentration percentages (A.C. %) of the elements present on the membrane surface as well as the O/Al ratio. These results show higher atomic concentration values of oxygen (theoretical (O/Al) ratio = 1.5), which is basically attributed to the H_3_PO_4_ aqueous solution employed for the final opening of the membrane pores as it was already indicated and it is described in the Experimental Section (Item 3.1). In fact, if the phosphorous content detected is associated to the PO_4_^3^^−^ from the H_3_PO_4_ solution, the oxygen associated to the O/Al ratio is reduced and the corrected value of (O/Al)* = (48.7 − 8.0)/25.4 = 1.6 is practically similar to the theoretical value, taking into account the possible small contribution also from the incorporation of electrolyte anions (oxalates) into the structure of the anodic aluminium oxide. The presence of carbon is also associated with the incorporation of electrolyte anions to the anodic oxide, as well as possible environmental contamination [21].

**Table 1:** Average values of the atomic concentration percentages of the elements found on both surfaces of the Al-O*x* membrane.

Membrane	C 1s (%)	O 1s (%)	Al 2p (%)	P 2s (%)	(O/Al)
Al-O*x*	23.9	48.7	25.4	2.0	1.9

### 2.2. Diffusive Transport Analysis

The diffusive permeability (*P_s_*) of a membrane is determined from mass transport at steady-state measurements. The flux of solute through the membrane (*J_s_*) is related with the concentration gradient across the membrane or concentration difference between both membrane surfaces (∆*C* = *C_f_* − *C_r_*) [22]:

*J_s_* = *dn*/*dt* = *P_s_* (*C_f_* − *C_r_*) = *P_s_*∆*C* (1)

where *dn* represents the elemental molar mass crossing the membrane in time *dt*. Since *dn*/*dt* = (1/*V_o_*)(*dC*/*dt*), Equation (1) can also be expressed as:

*dC_r_*/(*C_f_* − *C_r_*) = (*S*/*V*)*P_s_dt* (2)

where *V* is the volume of the solution in the cell and *S* is the cross-section. Taking into account the continuity of mass: *C_f_*° + *C_r_*° = *C_f_*^t^ + *C_r_*^t^ = cte, where *C_f_*° and *C_r_*° indicate the feed and receiving initial concentrations (time *t* = 0), while *C_f_*^t^ and *C_r_*^t^ correspond to these concentrations at a certain time *t*, the following expression is obtained [23]:

*ln*([*∆C^t^*/*∆C_f_°*)] = −*2*(*S*/*V*).*P_s_.t* (3)

**Figure 2:**
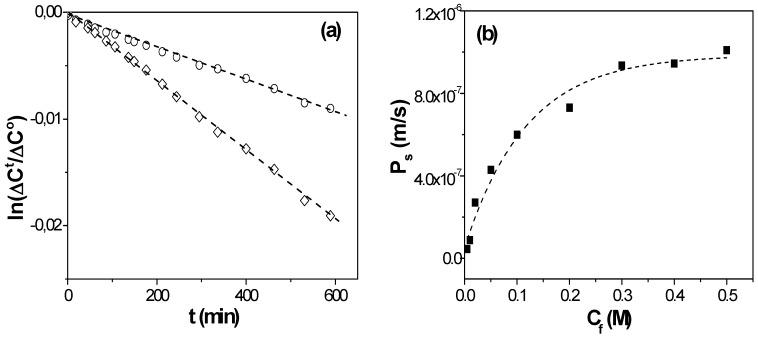
(**a**) Time evolution of solutions concentrations for *C_f_* = 0.01 M (o) and *C_f_* = 0.1 M (◊); (**b**) Variation of diffusive permeability in the membrane with NaCl feed concentration.

Figure 2a shows time evolution of NaCl concentration in feed and receiving solutions separated by the NPAM membrane for two different feed NaCl concentrations (*C_f_* = 0.05 M and *C_f_* = 0.3 M), and the diffusive permeability in the membrane at each *C_f_* value was obtained by the slope of each straight-line according to Equation (3). Variation of *P_s_* values with the feed concentration is shown in Figure 2b. The *P_s_*-*C* curve obtained for the NPAM shows two zones clearly differentiated related with the predominance of electrical (1) or diffusive (2) effects. The electrical electrolyte-membrane interactions seems to have a significant contribution at low feed concentrations (zone (1), for *C_f_* < 0.1 M NaCl), when the value of the effective fixed charge in the membrane (*X_ef_*) is lower than the electrolyte concentration; however, at high salt concentration (*C_f_* > 0.1 M NaCl, zone (2)) an almost constant value for *P_s_* was reached, which is associated with the increase of the screening effect of the higher number of electrolyte charges on the membrane fixed charge, with the consequent increase of diffusive transport contribution.

Filippov *et al.* [20] proposed an expression for the *P_s_*-*C_f_* curve for dense membranes, which considers both electrical and diffusive effects that, for porous membranes, is given by:

*P_s_ = (D_s_Θ|X_ef_|/2C_f_∆x)([1 + (2C_f_/|X_ef_|))^2^] ^1/2^ − 1)* (4)

where ∆*x* and *Θ* represent the pore length (membrane thickness in the case of straight pores perpendicular to the membrane surface) and membrane porosity, respectively, while *D_s_* is the diffusion coefficient of NaCl in the membrane nanopores. As it can be observed in Equation (4), diffusive transport depends on membrane geometrical parameters and, consequently, their correct evaluation is a basic point for an adequate determination of *P_s_* and *X_ef_* values, directly related with the mass/charge transport through the nanopores of the alumina membrane. The fitting of the data-point shown in Figure 2b allows us to estimate the following values for |*X_ef_*| = 0.012 M and *D_s_* = 4.7 × 10^−10^ m^2^/s, which hardly differ from those previously obtained from different experiments and models (Teorell-Meyer-Sievers model for membrane potential [24]) [25]. A comparison of the NaCl diffusion coefficient value in the membrane and in solution (*D_NaCl_*° = 1.61 × 10^−9^ m^2^/s [26]) shows a reduction of ~70% in the diffusive transport of NaCl, which is basically due to the confinement in a charged nanopore. The value of the diffusion coefficient is in good agreement with those previously reported in the literature for nanoporous alumina membranes with pore radii of 11 nm, 16 nm, and 120 nm (*D_s_* = 3.4 × 10^−10^ m^2^/s, 5.4 × 10^−10^ m^2^/s and 14 × 10^−10^ m^2^/s, respectively) obtained from membrane potential measurements [9,14], showing the correlation between these parameters.

**Figure 3:**
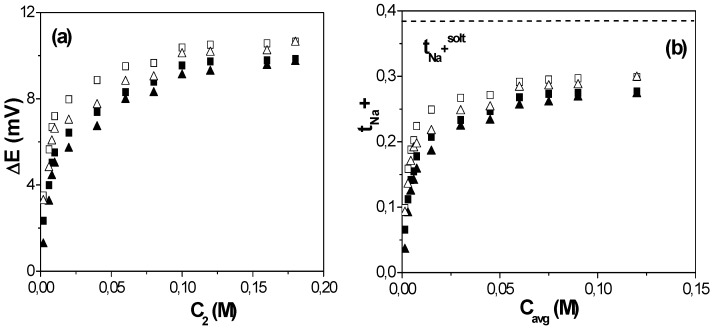
(**a**) Concentration potentials *versus* C_2_ solution concentration for the studied Al-O*x* NPAM for both opposite external and solution stirring conditions; (**b**) Cation (*t*_+_ = *t*_Na+_) transport number as a function of the average concentration C_avg_. Dense symbols: stirred solutions; open symbols: non-stirred solutions. Solution cation transport number: dot-dashed line.

The presence of fixed charges in the structure of a membrane is a factor that can significantly affect the transport of electrolyte solutions and charged species, and it is usually studied by measuring concentration potentials [27]. Figure 3a shows the variation of the concentration potential (∆*E*) as a function of the low NaCl concentration (C_2_) determined for both stirred and non-stirred solutions measurements as well as opposite situations (C_2_ in contact with each membrane surface). As it can be observed, in both cases these results also show two branches with rather different slopes depending on the concentration values, while the similarity of ∆*E* values for the opposite external conditions can be considered as an indication of membrane symmetry.

The effective fixed charge, *X_ef_*, and the transport number of the ions in the membrane, *t_i_*, are the two parameters usually determined when the diffusive transport of electrolyte solutions in membranes is analyzed [24]. The ion transport number represents the fraction of the total current transported for one ion (*t_i_* = *I_i_*/*I_T_*, with *t_+_* + *t_-_* = 1 for single salts), and consequently, the presence of charges on both external surface and pore wall (or internal surface) can significantly affect the value of *t_i_*, by rejecting ions with the same sign as the membrane charge and favoring the transport of the counter-ions [24]. Thermodynamic arguments lead to the following relationship between the measured electromotive force or concentration potential ∆*E* and the average value of the cation transport number *t_+_* for a pair (C_1_, C_2_) of solution concentrations (for 1:1 electrolytes) [24]:

∆*E* = −(2RT/F) *t*_+_ (ln(C_1_/C_2_) (5)

where R and F are the gas and Faraday constants, and T is the thermodynamic temperature of the system. For an ideal cation-exchanger membrane, *t_+_* = 1 and the concentration potential reaches its maximum value for the given concentration ratio [24]:

∆*E*_max_ = −(2RT/F) ln(C_1_/C_2_) (6)

Then, the value of *t*_+_ for each pair of solution concentrations can be obtained as [24,28]: *t*_+_ = ∆*E*/∆*E*_max_, while the anion transport number is *t_−_* = 1 – *t_+_*. Figure 3b shows the variation of cation transport number with the average solution concentration, C_avg_ = (C_1_ + C_2_)/2, for the studied Al-O*x* NPAM; for comparison, the value of the cation transport number in the solution is also indicated (dot-dashed line). These results clearly show the electropositive character of the Al-O*x* NPAM, with *t*_Na+_ < *t*_Na+_^solution^ = 0.385 [26], and the significant contribution of the Donnan exclusion of the co-ion (cation) at solution concentrations lower than the membrane fixed charge. However, the exclusion associated to solute-membrane electrical interactions is reduced at high concentrations, and almost constant values for *t*_Na+_ in the membrane pores are obtained.

Another point of interest is the difference found between stirred and non-stirred concentration values (dense and open symbols in Figure 3b, respectively), which is related with the barrier behavior of the membrane, and it consequently modifies the concentration values at the membrane-solution interface with respect to bulk solutions. Variations in *t_i_* values of around 25% were obtained at low NaCl concentrations depending on solution hydrodynamic conditions, but they reduce to 10% at high solution concentrations. These results clearly show the importance of interfacial effects for an adequate determination of transport parameters through nanopores or nanochannels since they might mask the real values.

## 3. Experimental Section

### 3.1. Synthesis of the Nanoporous Alumina Membrane

A highly ordered NPAM with hexagonally distributed pore arrangement was prepared by the two-step anodization of aluminum [10,29]. Firstly, high purity aluminum foil substrate (99.999%, Goodfellow, Huntingdon, England), with 0.5 mm in thickness and 25 × 25 mm^2^ in area was cleaned by sonication for 5 min in isopropanol and ethanol and then rinsed with deionized water. The Al substrate was then loaded in a two-electrode electrochemical cell built in Teflon and equipped with a thermally and electrically conductive Cu base and a mechanical stirring system. The cathode electrode was formed by a platinum wire net, while the aluminum substrate was used as anodic electrode in the following electrochemical processing steps. In order to obtain a smooth surface, the Al substrate was electropolished under an applied constant potential of 20 V in a mixture of perchloric acid and ethanol (1:3 in vol.) at 5 °C, which was stirred during the whole process. The two-step anodization process was then performed in oxalic acid 0.3 M aqueous electrolyte, at 1–3 °C and under an anodization potential of 40 V. The first anodization step lasted for 72 h to ensure a high ordering degree of the nanopores spatial distribution over the sample surface. The aluminum oxide layer grown in this first anodization step was selectively removed by wet chemical etching performed for 12 h in a solution composed of CrO_3_ and H_3_PO_4_ and kept at 45 °C. The second anodization step was performed under the same conditions as the first one, but the duration was adjusted to 35 h, in order to obtain a membrane thickness of around 60 µm.

To obtain a nanoporous alumina membrane suitable for flow-through experiments, the remaining Al substrate not consumed after the two-step anodization process was selectively removed by immersing the sample in CuCl_2_ and HCl aqueous solution at 20 °C. Finally, the alumina barrier layer that closes the pore bottoms was removed by floating the sample in 10 wt % H_3_PO_4_ acid solution, until the sample was totally immersed into the solution, due to the flow of the etching solution through the pore channels. The so obtained NPAM will be hereafter called as Al-O*x*.

### 3.2. Morphological Characterizations

The morphology of NPAM was studied by top, bottom, and cross-view section scanning electron micrographs, SEM, obtained in a JEOL–6100 microscope (JEOL Ltd., Tokyo, Japan) operating at 20 kV. Prior to SEM imaging, the sample surfaces were coated with a thin gold layer deposited by sputtering (Polaron SC7620 Sputter Coater, Quorum Technologies, East Sussex, England), in order to enhance the electrical conductivity of the samples, thus avoiding undesired charging effects upon electron beam irradiation.

The displayed values of pore radii and average interpore distance were obtained from SEM images by image analysis employing Image J (version 1.48v) software (National Institutes of Health, Bethesda, MD, USA) [30].

### 3.3. XPS Measurements

The surface of the studied membrane was chemical characterised by XPS. For these measurements a Physical Electronics spectrometer (PHI 5700, Chanhassen, MN, USA) equipped with X-ray Mg K_α_ radiation (300 W, 15 kV, 1253.6 eV) as excitation source was used. High-resolution spectra were recorded at a constant value of the take-off angle (45°) by using a concentric hemispherical analyzer, which operates in the constant pass energy mode at 29.35 eV. The residual pressure in the analysis chamber was maintained below 5 × 10^−7^ Pa during the acquisition of data and the diameter of the analysis area was 720 μm [31]. Binding energies were determined with respect to the position of the adventitious C 1s peak at 285.0 eV with an accurate ±0.1 eV. The software package of PHI ACCESS ESCA-V6.0 F (Chanhassen, MN, USA) was used for data acquisition and analysis.

### 3.4. Salt Diffusion and Concentration Potential Measurements

For membrane electrochemical characterization salt diffusion and concentration potential measurements were performed in dead-end test cells similar to that described in reference [32]. The membrane was placed in the middle of two symmetric half-cells separating two different solutions. Two Teflon-covered magnetic stirrers with a stirring rate of 540 rpm were used to minimize concentration-polarization at the membrane/solution interfaces [33]. Measurements were carried out at room temperature (25 ± 2) °C and the solution’s standard pH (5.8 ± 0.3).

NaCl diffusion measurements were performed with the membrane separating feed (*C_f_*) and receiving (*C_r_*) solutions. Time variation in the conductivies of both solutions (σ_f_ and σ_r_, respectively) were recorded with two conductivity cells connected to a digital conductivity meter (Crison GLP 31) placed each one in each half-cell. Calibration curve (NaCl solution conductivity *versus* concentration) was used for solution concentration determination. Salt diffusion measurements were carried out at different feed concentrations: *C_f_* (M) = 0.005, 0.01, 0.02, 0.05, 0.1, 0.25, and 0.4 NaCl.

The concentration potential or electromotive force (∆*E*) measured between two NaCl solutions of different concentrations (C_1_ and C_2_) placed at both sides of the membrane was measured by inserting two reversible Ag/AgCl electrodes (one in each half-cell), which were connected to a digital voltmeter (Yokohama 7552, 1GΩ input resistance, Tokyo, Japan). Measurements were performed keeping constant the concentration ratio of the solutions at both sides of the membrane, *r* = C_1_/C_2_ = 2, with solution C_1_ ranging between 0.002 M and 0.2, for both opposite situations (C_1_ in contact with top or bottom membrane surfaces) in order to check their similarity. Moreover, to see the effect of concentration polarization on concentration potential, value measurements were performed without stirring the solutions.

## 4. Conclusions

Manufacture, geometrical, and surface chemical characterization of a nanoporous alumina membrane is described. Membrane material but also chemical treatments associated with membrane manufacture might affect the membrane behavior. In this context, the work also shows the significant effect of the electrical interactions in the diffusive transport of electrolytes solutions (charged species in general) across NPAMs at low salt concentrations, although they can be practically neglected for concentrations higher than the effective fixed charge in the membrane (*X_ef_*).

Particularly, for a nanoporous alumina membrane obtained from the two-step anodization method in oxalic acid electrolyte, with an average pore radii of 23 ± 3 nm and porosity of 20% ± 1%, the estimated value for the effective fixed charge is *X_ef_* = 0.012 M, while the NaCl diffusion coefficient in the nanopores is *D_s_* = 4.7 × 10^−10^ m^2^/s, which represents a reduction of ~70% with respect to its value in solution. Moreover, as a result of the electropositive character of the Al-O*x* NPAM, a significant reduction in the transport of Na^+^ ions at low concentrations was found, but an average value for the Na^+^ transport number of 0.33 ± 0.01 was obtained for NaCl concentrations higher than *X_ef_*, which represents a reduction of ~15% with respect to solution. These results are in good agreement with previously reported values obtained from membrane potential measurements.

Consequently, information on membrane fixed charge gives valuable information for the election of the most adequate concentration related with the transport of electrolytes or charged solutions through NPAMs, which is a key parameter for the correct characterization of their diffusive ion transport behavior.
